# Supplementary material for: Varying Opinions about Animal Welfare in the Australian Live Export Industry: A Survey
Source: Animals (Basel). 2020 Oct 13;10(10):1864. doi: 10.3390/ani10101864 (PMC7602067; doi:10.3390/ani10101864)
Supplement: Supplementary file 1 [file animals-10-01864-s001.pdf]

Welfare Indicators

Hello and welcome

Page description:

Unique ID **Action: Hidden Value**
Value: [survey("counter"), startat="WI01"],

ID 270

Hello,
I am an independent researcher at Murdoch University in Perth, Western Australia, and I am undertaking a research project, funded by Meat and Livestock Australia, to develop key animal welfare measures for the livestock export industry, that:

- are informative about an animals' welfare
- are important to stakeholders (you)
- are practical for assessing livestock (sheep, cattle and goats) throughout the live export supply chain
- are economical, and
- are measurable and quantifiable

We want to know what you think about animal welfare in the live export industry, and what you want.
You have been sent this survey because you either work within the live export industry, or you have an interest in the industry, in particular animal welfare within the industry.

This survey will take approximately 30 minutes to complete.
You can save your progress and continue at a later stage by clicking 'save and continue' at the top of the screen at any point. Please remember to come back and complete the survey because your participation is important to us.

If you are uncomfortable completing the survey online, we can arrange for you to complete the survey by telephone.

We appreciate your time to complete this survey, and ask that you complete the whole survey as accurately and honestly as possible.
Information collected from this survey will be analysed and used to recommend welfare

measures to Meat and Livestock Australia and the livestock export industry.
Your privacy is very important to us. Your participation in this study and any information will be treated in a confidential manner.

This study has been approved by the Murdoch University Human Research Ethics Committee (Approval 2015/013). If you have any reservation or complaint about the ethical conduct of this research, and wish to talk with an independent person, you may contact Murdoch University's Research Ethics Office (Tel. 08 9360 6677 (for overseas studies, +61 8 9360 6677) or e-mail ethics@murdoch.edu.au). Any issues you raise will be treated in confidence and investigated fully, and you will be informed of the outcome.

If you have any questions at any time during the completion of this survey, or would like to receive a copy of the results once the study is complete (estimated completion time January 2016) please send me an email at AWResearch@murdoch.edu.au
Results will also be available on our facebook page https://www.facebook.com/animalwelfareresearch at the completion of the study.

There are comment boxes throughout the survey, if you wish to leave comments as you go.

Thank you

ID 756

1. **CONSENT**

1. I agree voluntarily to take part in this study.
2. I have read the Information Sheet provided and been given a full explanation of the purpose of this study, the procedures involved and of what is expected of me.
3. I understand that I will be asked to complete a survey
4. The researcher has answered all my questions and has explained possible problems that may arise as a result of my participation in this study.
5. I understand I am free to withdraw from the study at any time without needing to give any reason.
6. I understand I will not be identified in any publication arising out of this study.
7. I understand that my name and identity will be not be collected.
8. I understand that all information provided by me is treated as confidential and will not be released by the researcher to a third party unless required to do so by law.

Do you consent to participate in this survey? *

○ Yes, I consent to participate in this survey

Demographics

Page description:

ID 271

The questions in this section all relate to your background.
All information collected is kept confidential and will not be released to any third party.

ID 3

2. What is your gender?

- Male
- Female

ID 4

3. What age category are you?

- 18-21
- 21-30
- 31-40
- 41-50
- 51-60
- 61-70
- >71

ID 390

4. Where were you born?

- Australia
- New Zealand / South Pacific
- Middle East
- Europe
- Asia
- Africa
- North America
- South America
- Other

*

LOGIC Show/hide trigger exists.
ID 391
5. Where are you currently based for work?
If you are not currently working, please select the location of your last employment.

- Australia
- New Zealand / South Pacific
- Middle East
- Europe
- Asia
- Africa
- North America
- South America
- Other

*

LOGIC Hidden unless: Question "Where are you currently based for work?
If you are not currently working, please select the location of your last employment." #5 is one of the following answers ("Australia")

ID 392

6. In which State or Territory are you based for work?

- WA
- SA
- NSW
- QLD
- NT
- TAS
- ACT
- VIC

ID 96

7. Where have you lived most of your life?

- Rural / countryside only
- City/town only
- Mainly rural / countryside, some city/town
- Mainly city/town, some rural / countryside

ID 21

8. What is your highest level of education?

- Did not complete high school
- Graduated high school or equivalent
- Post-secondary school qualification (e.g. diploma)
- Undergraduate degree
- Post-graduate degree (e.g. Masters, PhD)

VALIDATION Min = 0 Max = 100

ID 496

9. Rate how important animals are to you in the following roles

	Not important	Important
As food		
As clothing		
As a companion		
For working		
For entertainment (i.e. racing)		
For performance (i.e. circus)		
As a religious symbol		

Comments

Stakeholder group

Page description:

ID 272

This section is to determine your connection with the livestock export industry

LOGIC Show/hide trigger exists.

ID 66

10. Do you work with the live export industry (i.e. employed, supply, inspect, research)? *

- Yes
- No

LOGIC Show/hide trigger exists. Hidden unless: Question "Do you work with the live export industry (i.e. employed, supply, inspect, research)?" #10 is one of the following answers ("Yes")

ID 519

11. In which area of the livestock export industry to you work most?

- Producer / supplier
- Transport (truck, ship, aeroplane)
- Export
- Abattoir
- Inspection / research

LOGIC Hidden unless: Question "In which area of the livestock export industry to you work most?" #11 is one of the following answers ("Transport (truck, ship, aeroplane)")

ID 521

12. In which transport areas do you work?

- ☐ Truck company owner / driver
- ☐ Ship owner / Captain
- ☐ Aeroplane owner / pilot
- ☐ Stockperson (ship)
- ☐ Airport freight
- ☐ At the sea port
- ☐ Administration

LOGIC Hidden unless: Question "In which area of the livestock export industry to you work most?" #11 is one of the following answers ("Export")

ID 525

13. In which export areas do you work?

- ☐ Stockperson at a feedlot
- ☐ Pre-export assembly depot manager
- ☐ Exporter
- ☐ Administration / management

LOGIC Hidden unless: Question "In which area of the livestock export industry to you work most?" #11 is one of the following answers ("Inspection / research")

ID 524

14. In which areas do you inspect / research?

- [ ] Truck transport
- [ ] Feedlot (in Australia)
- [ ] Sea or air port (In Australia)
- [ ] On-board ship
- [ ] On-board aeroplane
- [ ] Feedlot (not in Australia)
- [ ] Abattoir (not in Australia)

(untitled)

Page entry logic:
This page will show when: Question "Do you work with the live export industry (i.e. employed, supply, inspect, research)?" #10 is one of the following answers ("Yes")

Page description:

VALIDATION Min = 0 Max = 100

ID 17

15. What percentage of your work is associated with each of the following species? e.g. cattle: 50%, sheep: 40%, goats: 10%.

	0 %										100 %
	0	10	20	30	40	50	60	70	80	90	100
Cattle											
Sheep											
Goats											

Comments

LOGIC Show/hide trigger exists.

ID 601

16. Have you ever visited the following? Tick all that apply.

- ☐ Feedlot
- ☐ A live export ship or the port
- ☐ An aircraft carrying livestock
- ☐ Abattoir
- ☐ None of the above

ID 602

17. How many times have you visited each of the following locations?

Cattle Sheep Goats

Comments

ID 685

17. On average, how often do you see livestock being transported by truck?

Cattle
- Daily
- Once a week
- Once a fortnight
- Once a month
- Once every 6 months

Sheep
- Daily
- Once a week
- Once a fortnight
- Once a month
- Once every 6 months

Goats
- Daily
- Once a week
- Once a fortnight
- Once a month
- Once every 6 months

Comments

Experience

Page entry logic:
This page will show when: Question "Do you work with the live export industry (i.e. employed, supply, inspect, research)?" #10 is one of the following answers ("No")

Page description:

ID 72

19. What is your interest in the live export industry?
Please select all that apply

- ☐ Relative/friend works in the live export industry
- ☐ Concern for animals
- ☐ Animal welfare advocate
- ☐ Researcher/scientist
- ☐ Journalist/writer/media
- ☐ Don't care about it
- ☐ Other

LOGIC Show/hide trigger exists.

ID 615

20. Have you ever visited or seen the following? Tick all that apply.

- ☐ Livestock being transported by truck
- ☐ Feedlot
- ☐ A live export ship or the port
- ☐ An aircraft carrying livestock
- ☐ Abattoir
- ☐ None of the above

ID 616

21. How many times have you visited each of the following locations?

Cattle	Sheep	Goats

Comments

ID 699

21. On average, how often do you see livestock being transported by truck?

Cattle
- Daily
- Once a week
- Once a fortnight
- Once a month
- Once every 6 months

Sheep
- Daily
- Once a week
- Once a fortnight
- Once a month
- Once every 6 months

Goats
- Daily
- Once a week
- Once a fortnight
- Once a month
- Once every 6 months

Comments

Opinions

Page description:

ID 299

This section addresses what you think about animal welfare in the livestock export industry.

ID 117

23. What is your source of knowledge of the livestock export industry?

- [ ] Journalism media (print, television, internet)
- [ ] Social media (Facebook, Twitter etc...)
- [ ] Personal experience
- [ ] Chatting to friends
- [ ] Scientific journals
- [ ] Other

ID 765

24. What is your opinion of the livestock export industry as it is today? Please indicate for each species

	Continue as is	Continue with improvements	Should stop	Undecided
Cattle	○	○	○	○
Sheep	○	○	○	○
Goats	○	○	○	○

Comments

ID 503

25. Do you have concerns about the welfare of animals during the livestock export process?

	Cattle	Sheep	Goats
During truck transport not in Australia	Sometimes<br>Frequently<br>Always	Sometimes<br>Frequently<br>Always	Sometimes<br>Frequently<br>Always
At the feedlot in Australia	Sometimes<br>Frequently<br>Always	Sometimes<br>Frequently<br>Always	Sometimes<br>Frequently<br>Always
During loading/unloading at the port (sea)	Sometimes<br>Frequently<br>Always	Sometimes<br>Frequently<br>Always	Sometimes<br>Frequently<br>Always
On-board ship during a voyage	Sometimes<br>Frequently<br>Always	Sometimes<br>Frequently<br>Always	Sometimes<br>Frequently<br>Always
On-board aeroplane during a flight	Sometimes<br>Frequently<br>Always	Sometimes<br>Frequently<br>Always	Sometimes<br>Frequently<br>Always
At the feedlot not in Australia	Sometimes<br>Frequently<br>Always	Sometimes<br>Frequently<br>Always	Sometimes<br>Frequently<br>Always
During slaughter (not in Australia)	Sometimes<br>Frequently<br>Always	Sometimes<br>Frequently<br>Always	Sometimes<br>Frequently<br>Always

Comments

(untitled)

ID 481

Now we are going to give you some examples of animal welfare measures and ask your opinions on them.

ID 705

26. Which of the following indicators do you think are important when assessing animal welfare?

Description of welfare indictors
*Behaviour means how an animal is expressing itself, or refers to what an animal is doing
**Body condition score refers to how much fat is on an animal, whereas body weight encompasses the whole animal
***Respiration rate and character refers to how quickly and how easily an animal is breathing
****Rumination rate refers to how quickly food moves through the digestive system

	Do you think it is important to measure?			Do you think it is practical to measure?		
	Yes	No	Don't know	Yes	No	Don't know
Behaviour	○	○	○	○	○	○
Body condition score	○	○	○	○	○	○
Body temperature	○	○	○	○	○	○
Body weight	○	○	○	○	○	○
Death	○	○	○	○	○	○
Disease / health	○	○	○	○	○	○
Heart rate	○	○	○	○	○	○
Physiological status (i.e. pregnant / lactating)	○	○	○	○	○	○
Rumination rate	○	○	○	○	○	○
Respiration rate and character	○	○	○	○	○	○
Stress hormones	○	○	○	○	○	○
Wool length	○	○	○	○	○	○
Meat quality	○	○	○	○	○	○

Comments

(untitled)

ID 742

27. Which of the following **health** measures do you think are important when assessing animal welfare?

	Do you think it is important to measure?			Do you think it is practical to measure?		
	Yes	No	Don't know	Yes	No	Don't know
Presence of disease	○	○	○	○	○	○
Parasites	○	○	○	○	○	○
Infection	○	○	○	○	○	○
Injury/wounds	○	○	○	○	○	○
Lameness	○	○	○	○	○	○
Pain	○	○	○	○	○	○
Sneezing	○	○	○	○	○	○
Coughing	○	○	○	○	○	○
Nasal discharge	○	○	○	○	○	○
Faeces structure	○	○	○	○	○	○
Inability to stand	○	○	○	○	○	○
Vocalisations	○	○	○	○	○	○

Comments

(untitled)

ID 748

28. Which of the following **environmental** measures do you think are important when assessing animal welfare?

	Do you think it is important to measure?			Do you think it is practical to measure?		
	Yes	No	Don't know	Yes	No	Don't know
Smell	○	○	○	○	○	○
Amount of ventilation	○	○	○	○	○	○
Air quality	○	○	○	○	○	○
Amount of shade	○	○	○	○	○	○
Amount of shelter from weather	○	○	○	○	○	○
Humidity	○	○	○	○	○	○
Air temperature	○	○	○	○	○	○
Daily amount of light	○	○	○	○	○	○
Noise	○	○	○	○	○	○
Weather	○	○	○	○	○	○

Comments

(untitled)

VALIDATION Min = 0 Max = 100

ID 733

29. How important is the time an animal spends at any one point along the live export chain?

	Not important	Important
During truck transport		
On-board a truck waiting to unload		
On-board ship docked at port		
On-board ship during a voyage		
On-board an aircraft during a flight		
At a feedlot in Australia		
At a feedlot not in Australia		

Comments

(untitled)

VALIDATION Min = 0 Max = 100

ID 666

30. How important do you think the following factors are for the welfare of livestock in the live export industry?

	Not important	Important
Vaccination status of the animal		
Access to a vet		
Design of facilities		
Cleanliness of facilities		
Competency of stockperson		
Attitude of stockpersons		
Animals can be traced from farm to slaughter		
Suitability of species to the environment		
Keeping animals in familiar social groups together		
Animals with horns housed with animals without		

horns

Amount of space the animals have

Access to bedding

Use of an electric prod

Use of a working dog

Type of food available

Feed quality

Water quality

Temperature of drinking water

Amount of trough space

Identifying animals that don't eat

Time off feed

Time off water

Transport conditions

Truck driver behaviour

Appropriate restraint during slaughter

Effective slaughter procedures

Comments

(untitled)

ID 752

31. What do you think measuring animal welfare should be used for? Tick all that apply.

- [ ] The industry to self regulate
- [ ] The government to regulate the industry
- [ ] To impose penalties and restrictions for poor welfare
- [ ] To award incentives for good welfare
- [ ] Highlight areas for research
- [ ] Highlight areas for investment in welfare improvement
- [ ] Provide measures of performance to the general public
- [ ] Not used for any reason
- [ ] Other

*

753

32. Who do you think should be monitoring and measuring animal welfare? Tick all that apply.

- [ ] Stockpersons
- [ ] Veterinarians that work for the live export industry
- [ ] Veterinarians that work for the Australian government
- [ ] Veterinarians that work for an overseas government
- [ ] Animal welfare inspectors
- [ ] Independent welfare inspectors
- [ ] Exporters
- [ ] Abattoir animal welfare officers
- [ ] Other

*

754

33. Do you think that data collected on animal welfare should:

- ○ be confidential to the industry and government only
- ○ be available to the public

(untitled)

Page description:

LOGIC Show/hide trigger exists.

ID 680

34. Are there any other animal welfare measures that we have not mentioned that you think are important?

- Yes
- No

LOGIC Hidden unless: Question "Are there any other animal welfare measures that we have not mentioned that you think are important?" #34 is one of the following answers ("Yes")

ID 435

35. List any animal welfare indicators that we have not mentioned that you think are important?
Please list them in order of importance, with the indicator you think is most important as #1.

	Animal welfare indicator	Do you think it is practical to measure?	
		Yes	No
1		○	○
2		○	○
3		○	○
4		○	○
5		○	○
6		○	○
7		○	○
8		○	○
9		○	○
10		○	○

ID 769

Please press the submit button at the bottom of this page

Thank You!

ID 1

Thank you for taking our survey. Your response is very important to us.

Results of this survey will be used to recommend appropriate animal welfare measures to the livestock export industry.

If you have any questions or comments, you can email me at AWResearch@murdoch.edu.au

If you would like to recieve a copy of the results once the study is complete (estimated completion time January 2016) please send me an email at AWResearch@murdoch.edu.au
Results will also be available on our facebook page
https://www.facebook.com/animalwelfareresearch at the completion of the study.
